# Effects of primary care C-reactive protein point-of-care testing on antibiotic prescribing by general practice staff: pragmatic randomised controlled trial, England, 2016 and 2017

**DOI:** 10.2807/1560-7917.ES.2020.25.44.1900408

**Published:** 2020-11-05

**Authors:** Charlotte Victoria Eley, Anita Sharma, Hazel Lee, Andre Charlett, Rebecca Owens, Cliodna Ann Miriam McNulty

**Affiliations:** 1 Primary Care Unit, Public Health England, Gloucester, United Kingdom; 2 An NHS Clinical Commissioning Group, Greater Manchester, United Kingdom; 3 Statistics Unit, Public Health England, London, United Kingdom

**Keywords:** C-reactive protein, point-of-care test, randomised control trial, antibiotics, general practice

## Abstract

**Background:**

C-reactive protein (CRP) testing can be used as a point-of-care test (POCT) to guide antibiotic use for acute cough.

**Aim:**

We wanted to determine feasibility and effect of introducing CRP POCT in general practices in an area with high antibiotic prescribing for patients with acute cough and to evaluate patients’ views of the test.

**Methods:**

We used a McNulty–Zelen cluster pragmatic randomised controlled trial design in general practices in Northern England. Eight intervention practices accepted CRP testing and eight control practices maintained usual practice. Data collection included process evaluation, patient questionnaires, practice audit and antibiotic prescribing data.

**Results:**

Eight practices with over 47,000 patient population undertook 268 CRP tests over 6 months: 78% of patients had a CRP < 20 mg/L, 20% CRP 20–100 mg/L and 2% CRP > 100 mg/L, where 90%, 22% and 100%, respectively, followed National Institute for Health and Care Excellence (NICE) antibiotic prescribing guidance. Patients reported that CRP testing was comfortable (88%), convenient (84%), useful (92%) and explained well (85%). Patients believed CRP POCT aided clinical diagnosis, provided quick results and reduced unnecessary antibiotic use. Intervention practices had an estimated 21% reduction (95% confidence interval: 0.46–1.35) in the odds of prescribing for cough compared with the controls, a non-significant but clinically relevant reduction.

**Conclusions:**

In routine general practice, CRP POCT use was variable. Non-significant reductions in antibiotic prescribing may reflect small sample size due to non-use of tests. While CRP POCT may be useful, primary care staff need clearer CRP guidance and action planning according to NICE guidance.

## Introduction

Seventy to eighty per cent of all antibiotics in the United Kingdom (UK) are prescribed in the community [1] and 60% of these antibiotics are issued for respiratory tract infections (RTI) [2]; 20% are thought to be unnecessary or inappropriate [3] as research suggests that acute RTI are often viral and do not require an antibiotic [2,4,5]. Reducing inappropriate antibiotic prescribing is fundamental to tackling antimicrobial resistance and The UK Five Year Antimicrobial Resistance Strategy aims to optimise prescribing practice by promoting better use of existing diagnostics [6].

The Lord O’Neill report, tackling drug-resistant infections globally, recommends that by 2020 it should be mandatory that the prescription of antibiotics is informed by data and testing technology, such as a diagnostic test, wherever available and effective to support clinical judgment to prescribe [7].

The National Institute for Health and Care Excellence (NICE) incorporated C-reactive protein (CRP) point-of-care tests (POCT) into the diagnosis of pneumonia guidelines CG 191 (Box 1) [8] and CRP POCT is also included in the NICE acute cough summary for antimicrobial prescribing [9]. The NICE recommends that CRP POCT should be considered when a patient presents with symptoms of lower RTI, clinical assessment is inconclusive and there is uncertainty whether antibiotics should be prescribed [8]. Even though CRP POCT is recommended by NICE and has the potential to improve patient care, the uptake of CRP POCT across England has been very variable and CRP POCT is not extensively used in primary care [10].

Box 1 **NICE Guidance CG 191: Pneumonia in adults: diagnosis and management [8]**

**Presentation with lower respiratory tract infection**

For people presenting with symptoms of lower respiratory tract infection in primary care, consider a point-of-care C-reactive protein test if after clinical assessment a diagnosis of pneumonia has not been made and it is not clear whether antibiotics should be prescribed. Use the results of the C-reactive protein test to guide antibiotic prescribing in people without a clinical diagnosis of pneumonia as follows:

-  Do not routinely offer antibiotic therapy if the C-reactive protein concentration is less than 20 mg/L.
-  Consider a delayed antibiotic prescription (a prescription for use at a later date if symptoms worsen) if the C-reactive protein concentration is between 20 mg/L and 100 mg/L.
-  Offer antibiotic therapy if the C-reactive protein concentration is greater than 100 mg/L.

NICE: National Institute for Health and Care Excellence.

Systematic reviews, for RTI in general and lower RTI specifically, show the value of CRP POCT on reducing antibiotic prescriptions for RTI [11-14]. Huang et al. reported that CRP POCT significantly reduced antibiotic prescribing at the index consultation for patients with RTI [11]. A randomised control trial found that general practitioners (GPs) in the CRP test group prescribed significantly fewer antibiotics compared with the control group [15] and that patients in the CRP test group used fewer antibiotics than the control [16]. This research was conducted in research practices in the Netherlands and results may not be replicated in a non-research setting with normal primary care service provision in England. A small pilot study with 94 patients conducted within a single GP surgery in Wales found that the practice using CRP POCT had significantly reduced their antibiotic prescribing compared with other practices in the health board [17].

The use of CRP POCT for acute cough may be particularly valuable in areas with high antibiotic prescribing, but there is inconsistent CRP test use in such areas (e.g. Northern England) [18]. Therefore, the present study aimed to determine if the introduction of CRP POCT into non-research practices in a Clinical Commissioning Group (CCG) with high antibiotic prescribing was feasible, to explore whether CRP POCT was acceptable to patients, and whether provision of CRP POCT reduced prescribing for acute cough compared with controls. The study aimed to measure antibiotic use via enhanced retrospective audit using Read codes in intervention and control practices. The main difference between our study and previous CRP POCT research is that our study was based in real-life patient populations found in non-research clinical practices, aiming to reflect the true potential use of CRP POCT and their impact in primary care in England.

## Methods

### Design and setting

We performed a service evaluation using the McNulty–Zelen clustered randomised controlled trial (RCT) design [19] in practices within a high-prescribing CCG in Northern England. A CCG is a clinically led statutory National Health Service (NHS) body responsible for the planning and commissioning of healthcare services for their local area. In this design, practices were not aware that they were taking part in an RCT or that they had been randomly assigned to an intervention or control group; they only knew that they were being part of a pilot of CRP POC testing in their CCG. Control practices were not told about the trial. Consent was given by the CCG on the practices’ behalf. The study was not registered as a trial to keep the study masked to practices.

### Stratification and randomisation 

**Figure 1:**
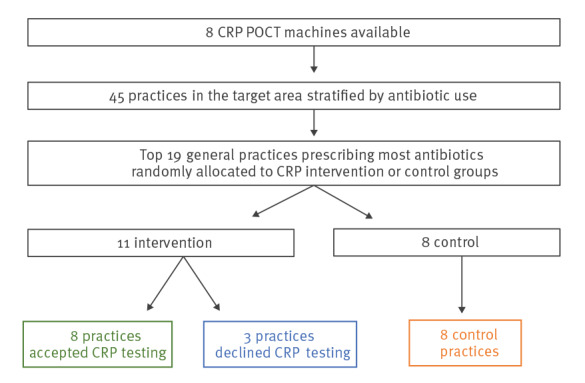
Recruitment flow chart, C-reactive protein point-of-care testing, Northern England, 2016–17 (n = 16 practices)

CRP: C-reactive protein; POCT: point-of-care test.

Forty-five general practices within a Northern England CCG were stratified by total antibiotic dispensing per 1,000 patients for 2016. The top 19 prescribers were randomly (using computer generated pseudo-random numbers) allocated to the intervention (offering CRP POCT) or control group (usual provision by the practice) (Figure 1).

In 2016, practices allocated to the intervention arm were offered a CRP POCT machine and up to 100 CRP tests to use over 6 months by a letter from the local GP antimicrobial stewardship (AMS) lead and follow-up phone call. Practices used the CRP POCT machine for 6 months between 1 August 2016 and 31 July 2017; start date was dependent on when the practices had received training. The study aimed for the use of 800 CRP POCTs to contribute to statistically significant results. Practices that agreed to take part in the study were visited by the AMS lead to promote the CRP POCT and received standard CRP POCT training by Alere Ltd (Stockport UK) which is usual practice when a diagnostic test is introduced into a laboratory or primary care setting. Alere was selected for this study as it was the most readily available CRP test in the UK at that time, had been reviewed by a Medtech innovation briefing [20] and was being used in other CCGs across England at that time [21]. The CRP testing kits were provided by Alere at cost to the CCG and were free to the healthcare staff. They comprised of the Alere Afinion CRP POCT manufactured by Alere Ltd. The test has a total assay time of 4 min on a sample volume of 1.5 µL capillary blood [22]. Alere were not involved in the planning of the study or interpretation of results, they only helped deliver the training to practices. 

### Patient inclusion/exclusion criteria

Practice staff were asked to offer patients over 18 years and under 65 years with acute cough a CRP POCT after clinical assessment and in accordance with national guidelines as appropriate [8] using patient selection criteria (Box 2). To avoid overuse of the test, clinicians using the CRP POCT were advised to use a diagnostic score to help them decide whether a CRP test was needed. The diagnostic score comprised: breathlessness, pulse > 100 bpm, temperature > 37.8 °C, crackles on the chest and diminished vesicular breathing and each symptom scored 1 point. A diagnostic score of at least 1 was advised before a CRP POCT should be considered. The controls did not use the diagnostic score as they did not know they were in the trial, this diagnostic score was only used by intervention practices. Use of the CRP POCT in practices stopped after a 6-month period or when the practice had used their allocated 100 CRP tests. Practices were asked not to use CRP testing for other clinical scenarios.

Box 2 **Study inclusion and exclusion criteria, C-reactive protein point-of-care testing, Northern England, 2016–17**

**Inclusion criteria**

- The patient is between 18 and 64 years inclusive.
- The patient has undergone clinical assessment (ideally using diagnostic score).
- The patient has given oral consent for the CRP test and understands the rationale for the test and process according to the clinician.
- The patient has a lower respiratory tract infection presenting diagnostic uncertainty.
- The presentation is acute (21 days or less from symptom onset).
- The patient has a primary complaint of cough.

**Exclusion criteria**

- The patient has a definitive indication for antibiotics (without diagnostic uncertainty), i.e. pneumonia.
- The patient is severely ill and definitely requiring antibiotics or hospital admission.

### Data collection

#### Patient descriptive data

We asked GP clinical staff to record on the clinical computer system routine clinical assessment, diagnosis, diagnostic score, CRP test result, antibiotic prescriptions (delayed if within 7 days of consultation or immediate). Patient re-consultations in the next 4 weeks and hospitalisation data were taken from routinely collected data on the practice clinical system. The patient descriptive data enabled us to determine if management of acute cough following a CRP POCT was in line with NICE guidance but was dependent on accurate inputting of patient records by staff. A medicine optimisation technician (author HL) visited each intervention practice to download this information from the EMIS Health general practice clinical data management system (https://www.emishealth.com). The EMIS Health clinical data management system supplies electronic patient record systems and software used in primary care, acute care and community pharmacy in the UK. Entry of NHS code/patient identifier was obligatory on the CRP POCT machine before each test and used to check patient computer records against NICE CRP guidelines.

#### Patient questionnaire

Patients were invited to complete a satisfaction questionnaire (Supplement S1) immediately after the CRP test or at home. Non-returns were reminded by letter and telephone call. 

#### Management of acute cough, bronchitis, chest and lower respiratory tract infection, and C-reactive protein test use in the practice using a Read code search

The Data Quality Team for Greater Manchester Shared Services hosted by an NHS CCG in Greater Manchester undertook an EMIS GP clinical system search to obtain diagnostic Read code and antibiotic prescribing data from intervention and control practices. The Read code search aimed to capture all patients presenting with acute cough, aged between 18 and 64 years inclusive, during the study period (1 August 2016–31 July 2017). To capture comparative data for the same 6 months in the previous year, the Read code data also included 12 months before the study (1 August 2015–31 July 2016). Patients presenting with acute cough as the main symptom fulfilled the inclusion criteria. However as clinicians have different clinical computer coding habits, to make sure all potential patients who had an acute cough were captured in the study, the data search included patients with acute cough, bronchitis, chest infection or lower RTI, which may all present with acute cough. The antibiotics included in this data collection were: amoxicillin, amoxicillin/clavulanic acid (search term used: co-amoxiclav), phenoxymethylpenicillin, doxycycline, tetracycline, oxytetracycline, clarithromycin, erythromycin and azithromycin.

There was an administrative merger between two of the intervention practices during the study. The consultation and prescribing data for these two practices were available as from a single provider, therefore in these analyses there are total of practices is only seven in the intervention arm.

### Data analysis

Descriptive analysis was conducted to describe: the total patient population, patients with acute cough, bronchitis, chest infection or lower RTI, GP trends in dispensing data for each GP practice in the period before and during the study, CRP data, practice Read code searches and closed patient questions. Authors CE and AC used Stata version 13.1 (StataCorp., College Station, United States) and visual graphs to represent the findings. Author CE analysed open-ended patient questions using NVivo software version 10 (Q S R International UK Ltd, Warrington, UK) to organise and code the data for thematic analysis. The main qualitative themes derived from the open-ended patient questions were discussed and agreed by the research team.

The primary objective was to determine if the intervention practices had reduced odds of prescribing antibiotics for lower RTI, bronchitis, chest infection and acute cough consultations compared with their prescribing practice during the same period in the previous year and compared with the controls during the same time periods. We used mixed-effects logistic regression models with the binary outcome of whether an antibiotic was dispensed or not. In each analysis, GP practice was included as a random intercept and dispensing in the same 6 months in the previous year, month, age and sex were included as fixed effects.

To assess the impact of the intervention, a mixed effects logistic regression model was conducted, with practice number and patient number as random effects; the random effect for patient was statistically unimportant, so only practice number was used as a random intercept. When there were multiple prescriptions for a single consultation these were combined such that the unit of analysis was a consultation, defined by practice code, EMIS number and date, and the binary outcome of no or at least one prescription for a consultation was used in the statistical model. Age, sex, proportion prescribed in the same 6 months in the previous year, month of prescription, diagnosis category, viral diagnosis, bacterial diagnosis and intervention were used as fixed effects in the regression model. An interaction between diagnosis category and intervention was also fitted to assess if there was any evidence that the intervention effect differed between the diagnosis categories.

### Ethical statement

National Research Ethics Committee approval was not required as the study did not recruit NHS patients, through the NHS. This decision is in accordance with the NHS ‘defining research’ guidelines [23]. The trial was approved by National Institute for Health Research Clinical Research Network Greater Manchester where the trial was taking place. Data were collected in line with the Data Protection Act 1998 and Caldicott 1999 regulations on handling and distributing sensitive participant information. All general practices provided written informed consent for their practice data to be extracted. Oral consent was obtained from patients for the CRP POCT and assumed for the patient questionnaire.

## Results

Of the 19 randomised practices, 11 were randomised to receive the CRP POCT machine; eight practices accepted and three declined. Following acceptance, eight practices were trained and six requested a second practice training visit. The number of CRP tests used in the eight practices ranged from 0 to 100 CRP POCT in the 6 months (median: 19.5).

### Study process evaluation

**Figure 2:**
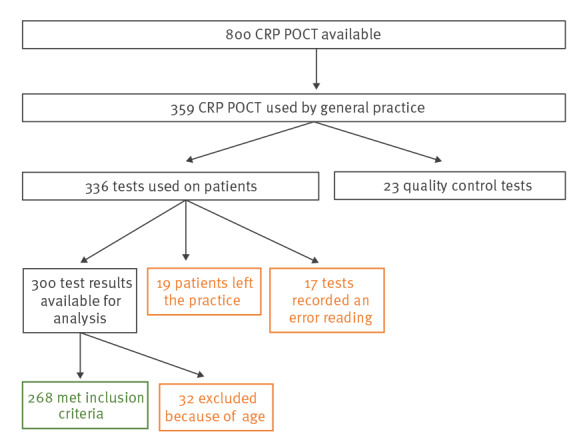
Data inclusion flow chart, C-reactive protein point-of-care testing, Northern England, 2016–17 (n = 8 practices)

CRP: C-reactive protein; POCT: point-of-care test.

Out of 800 tests allocated, 336 were used for patient testing and 23 were used as quality controls. Nineteen of the 336 patients tested left their practice so there were no patient data available, 17 had data input errors and 32 tests were undertaken in patients outside the age criteria in Box 2. Therefore, we included 268 of 336 patient CRP tests in the analysis (Figure 2).

The main presenting conditions were acute cough (57%; 153/268) or chest infection (24%; 64/268); other RTI presented included cold (6%; 16/268), sore throat (4%; 11/268), viral infection (1%; 3/268), ear pain (1%; 3/268), not recorded/other conditions (7%; 18/268). Overall CRP POCT uptake in the eight general practices ranged considerably dependent on number of consultations for 18–64-year-olds with lower RTI, bronchitis, acute cough and chest infection (Table 1).

**Table 1:** Use of C-reactive protein point-of-care testing in intervention practices, Northern England, 2016–17 (n = 268)

Practice	IA	IB	IC	ID	IE	IF	IG	IH	Totals
Usage rate (total CRP tests/registered patients)	1.5%	0.3%	0.05%	1.5%	0.6%	0.7%	0.6%	0%	0.6%
Pre-CRP assessment diagnostic score used^a^	41	9	1	62	0	10	93	NA	**216**
Number of CRP tests conducted that met inclusion criteria	70	5	3	61	21	14	94	0	**268**
Main CRP POCT user	GP	GP	GP/nurse	GP/nurse	Practice nurse	GP	Prescribing pharmacist	NA	NA
CRP POCT machine location	Nurses room	GP room	Nurses room	GP room	Clean store	Portable on a trolley	Pharmacist room	NA	NA
Number of consultations of 18–64 year-olds with LRTI, bronchitis, acute cough, chest infection^b^	182	84	292	40	204	100	284	Merged with intervention practice IC	1,186
CRP/100 consultations with LRTI, bronchitis, acute cough and chest infection	38.5	6.0	1.0	152.5^c^	10.3	14.0	33.1		22.6
Number of antibiotics^d^ prescribed on day of CRP test	110	43	169	19	104	74	118		637

CRP: C-reactive protein; GP: general practitioner; LRTI: lower respiratory tract infection; NA: not applicable; POCT: point-of-care test.

^a^ Clinical assessment score pre-CRP: breathlessness, pulse > 100, temperature > 37.8, crackles on the chest and diminished vesicular breathing.

^b^ Excludes upper respiratory tract infections, sinusitis, pharyngitis, laryngitis, tonsillitis, rhinitis/common cold, sore throat.

^c^ This figure is > 100 suggesting that CRP has been used for diagnoses outside of the four eligible diagnoses.

^d^ Total antibiotics (amoxicillin, amoxicillin/clavulanic acid, phenoxymethylpenicillin, doxycycline, tetracycline, oxytetracycline, clarithromycin, erythromycin and azithromycin).

**Figure 3:**
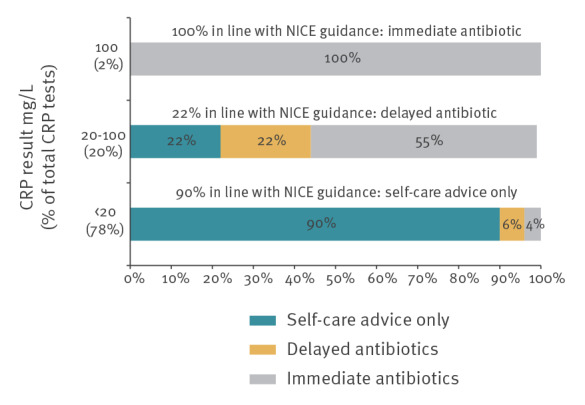
Summary of management actions following C-reactive protein point-of-care testing in line with NICE guidance [8], Northern England, 2016–17 (n = 268)

CRP: C-reactive protein; NICE: National Institute for Health and Care Excellence.

Most CRP test results were < 20 mg/L (78%; 209/268) and the management of these patients mainly followed NICE guidance to self-care and no antibiotics (90%; 188/209) (Box 1). All patients with a CRP > 100 mg/L were treated with immediate antibiotics (5/5) in line with NICE. However, only 12 of 54 with a CRP result between 20 and 100 mg/L were managed in line with NICE guidance which states that a delayed antibiotic prescription should be considered by the clinician (Figure 3).

Patients with a higher CRP test result were significantly more likely to re-consult in the next month: > 100 mg/L (2/5), 20–100 mg/L (8/55) and < 20 mg/L (23/208). The number of hospital admissions did not follow the same pattern. Of nine patients with a hospital admission (or complication that warranted further clinical examinations), five had a CRP test result < 20 mg/L, four had a CRP test result of 20–100 mg/L and none were > 100 mg/L.

A higher diagnostic score was associated with fewer patients with a CRP reading < 20mg/L. Among the 193 patients who had a diagnostic risk classification score before the CRP test, 106 (55%) had a diagnostic score of 0, 51 (26%) a score of 1, 26 (13% a score of 2, eight (4%) a score of 3, two (1%) a score of 4, and none had a score of 5. A CRP result < 20 mg/L was seen in 92 of the 106 with score 0, in 41 of 51 with score 1, in 17 of 26 with score 2, in two of eight with score 3, and in none of the patients with a score of 4.

### Patient views

**Figure 4:**
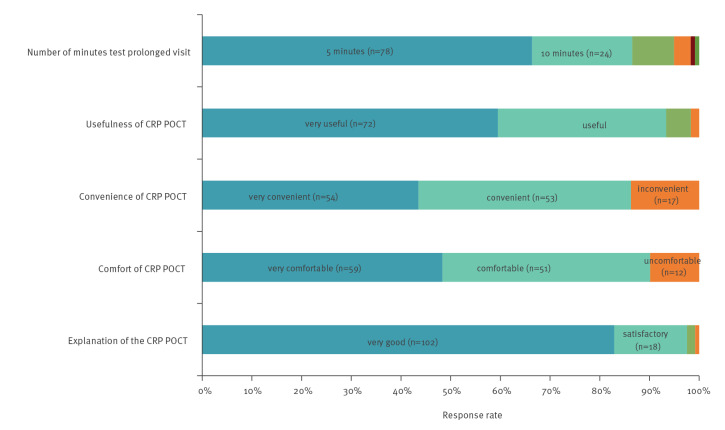
Patient feedback on C-reactive protein point-of-care testing, Northern England, 2016–17 (n = 134)

The patient satisfaction questionnaires were returned by 53% (134 of 251 distributed); 48 respondents were men (36%) and 82 women (61%), and 46 were completed on the day of the CRP test. For the individual questions, 48% (59/122) respondents described the CRP test as very comfortable, 44%(54/124) as very convenient, 60% (72/121) as very useful, 67% (78/116) reported that it prolonged their visit to the doctor by only 5 min, and 83% (102/123) reported that the explanation of the purpose of the test was very good (Figure 4).

Of the 122 patients who responded to this question, half reported that the test was conducted by prescribing pharmacists in the practice (60/122), 28% (34/122) by a GP, 18% (22/122) by a nurse and 5% (6/122) did not know. In the open ended questions, the most common comments were that the CRP test aids clinical diagnosis, provides quick results and reduces unnecessary antibiotic use (Table 2).

**Table 2:** Qualitative patient views on what they liked about the C-reactive protein point-of-care tests, Northern England, 2016–17 (n = 122)

Theme	Patient quotes
Aids clinical diagnosis	“Helps diagnosis and treatment” “Helped the doctor know whether I needed an antibiotic” “Diagnosed the problem there and then”
Provides quick results	I like that you “get an informative answer straight away” “It was good because it gave me immediate feedback” “Something quick and simple, easy to do and gave instant results”
Reduces unnecessary antibiotic use	“Saves issuing antibiotics when not needed” “Good for not giving antibiotics out if not needed” “Decides whether you need antibiotics or not, which is good if you need antibiotics and if you don’t need antibiotics. At least you know!”

Only four patients made negative comments about the CRP POCT: unsure if CRP test result was correct as they had to re-consult at the practice with worsening symptoms (n = 2), and the finger prick blood test was uncomfortable (n = 2). Most patients (78%; 101/130) stated they would definitely recommend that others who present with a cough should have a CRP test. Most would expect a CRP test when they next presented with an acute cough but it would depend on their symptoms (54%; 68/125), 93% (116/125) would accept a CRP test if their GP offered it and 78% (95/122) would be happy for a CRP test to be done at a local community pharmacy.

### Descriptive analysis: 6-month study trial

During the 6-month intervention there were 2,934 consultations (2,297 patients) for lower RTI , bronchitis, acute cough or chest infection, with 1,186 consultations (981 patients) in the intervention group and 1,748 (1,316 patients) in the control group. Nearly all antibiotics were prescribed on the consultation day (97%), with 12 deferred scripts Read-coded. A total of 654 (55.1%) of the consultations in the intervention arm had at least one antibiotic prescription, compared with 941 (53.8%) of consultations in the control arm that had at least one antibiotic prescription during the 6-month trial period.

In intervention and control practices, there was no evidence that prescribing differed between men and women, nor by the age of the patient. There were differences in the prescribing rate in both intervention and controls across the diagnosis categories, with a significantly higher prescribing rate for chest infections (n = 898), lower RTI (n = 456) and bronchitis (n = 39) compared with cough alone (n = 1,541) over the 6-month study period, chi square test of association 20.04, 1 degree of freedom p<0.001 (Table 3).

**Table 3:** Percentage of consultations with an antibiotic prescription, by diagnosis category, Northern England, 2016–17 (n = 1,595)

Diagnosis category	Practice					
	Control (n = 941)		Intervention (n = 654)		Total (n = 1,595)	
	n	%	n	%	n	%
Lower respiratory tract infection	229	65.5	227	71.7	456	68.4
Bronchitis	22	59.1	17	76.5	39	66.7
Chest/respiratory infection	550	78.5	348	82.2	898	80.0
Cough	947	36.5	594	32.9	1,541	35.0

### Statistical analysis: intervention versus control

**Figure 5:**
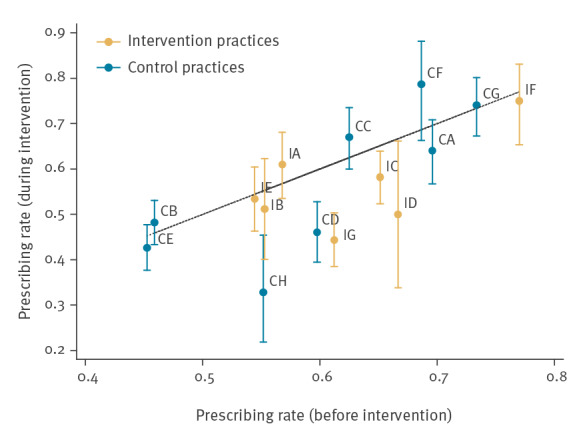
Antibiotic prescribing rate before and during the intervention period, C-reactive protein point-of-care testing, England, 2016–17 (n = 16 practices^a^)

^a^ Intervention practice IH was merged with IC during the study period.

Figure 5 shows that three intervention practices (IC, ID and IG) and two control practices (CD and CH) had significantly reduced antibiotic prescribing during the 6-month trial period compared with the same 6 months in the previous year. There were no practices that had significantly increased antibiotic prescribing; all other practices were similar during the 6-month trial period compared with the same 6 months in the previous year.

### Respiratory tract infection diagnoses 

A total of 2,934 consultations were used in the mixed-effects logistic regression analysis. There was an estimated 12% reduction in the odds of prescribing for these RTI diagnoses (including lower RTI, bronchitis, chest/respiratory infection and cough) in the intervention practices (95% confidence interval (CI): −34% to +16%) compared with the control practices, the model results are presented in Table 4.

**Table 4:** Estimated effect of C-reactive protein point-of-care testing on antibiotic use, mixed-effects logistic regression model including lower respiratory tract infection, bronchitis, chest/respiratory infection and cough, Northern England, 2016–17 (n = 2,934)

Predictor	Estimated OR	95% CI	p value
Intervention^a^	0.88	0.66–1.16	0.4
Baseline prescribing rate^b,c^	17.29	3.53–84.60	< 0.001
Age (per year)^b,d^	0.9935	0.9870–1.000	0.05
Sex (female)^e^	1.04	0.88–1.22	0.7
Diagnosis category			
Lower respiratory tract infection	Reference		
Bronchitis	0.83	0.40–1.70	0.6
Chest/respiratory infection	1.79	1.36–2.35	< 0.001
Cough	0.25	0.21–0.32	< 0.001

CI: confidence interval; OR: odds ratio.

^a^ Reference: control.

^b ^Continuous predictor in the range zero to one. The estimated OR is the relative change in odds for a theoretical unit change in the predictor.

^c^ Predictor included to account for baseline differences in pre-intervention prescribing.

^d^ Predictor included to account for change of one year of age.

^e ^Reference: male.

### Cough diagnoses

There were a total of 1,541 consultations with a diagnosis of cough over the 6-month trial. When considering just the cough diagnoses, there was a 21% reduction in the odds of prescribing in the intervention practices (estimated OR = 0.79; 95% CI: 0.46–1.35), however, the result was not statistically significant and could be due to chance alone (noted in Table 4 intervention; p = 0.4).

### Statistical analysis: high- versus low-fidelity practices

Three of the intervention practices, A, D and G, performed more than 60% of the tests available and more than 30 CRP tests per 100 consultations with lower RTI, bronchitis, acute cough and chest infection, and were classified as high-fidelity practices. The other, low-fidelity, practices undertook fewer than 15 tests per 100 consultations with lower RTI, bronchitis, acute cough and chest infection. In additional analyses, we further classified the binary variable of intervention or control into intervention (high fidelity), intervention (low fidelity) and control. After allowing for the other variables in the regression model, there was an estimated 19% reduction (95% CI: −17 to 34) in the odds of prescribing in the three high-fidelity practices (p = 0.26). In the intervention practices considered not to be high users of CRP POCT (low fidelity), there was an estimated 7% reduction (95% CI: −30 to 33) in the odds of prescribing (p = 0.7). Diagnoses relating to only cough saw a larger clinical reduction of 31% in the odds of prescribing (total antibiotics) in high-fidelity practices (Table 5).

**Table 5:** Estimated reduction in the odds of prescribing (total antibiotics) in intervention practices, compared to non-intervention practices, C-reactive protein point-of-care testing, Northern England, 2016–17 (n = 1,186)

	Estimated reduction in the odds of prescribing: total antibiotics (OR, 95% CI)		
Diagnosis included	All intervention practices	High-fidelity intervention practices	Low-fidelity intervention practices
Lower respiratory tract infection, bronchitis, chest/respiratory infection, cough (n = 1,186)	−12% (0.88; 95% CI: 0.66–1.16)	−19% (0.81; 95% CI: 0.66–1.17)	−7% (0.93; 95% CI: 0.67–1.30)
Cough (n = 594)	−21% (0.79; 95% CI: 0.46–1.35)	−31% (0.69; 95% CI: 0.35–1.38)	−13% (0.87; 95% CI: 0.46–1.65)

CI: confidence interval; OR: odds ratio.

Table 5 shows an estimated 19% reduction (95% CI: −17 to 34) in the odds of prescribing in the three high-fidelity intervention practices compared to non-intervention practices when considering the four diagnoses and a 31% reduction (95% CI: −38 to 65) when considering just cough diagnoses; both failed to reach statistical significance. Overall and for most subgroup of diagnoses, there were estimated reductions in the odds of prescribing in those three practices (high-fidelity) that performed most CRP POCT, particularly when considering just the cough diagnosis which was the intended patient group for this study. However, for none of these analyses did the reduction reach levels that would be considered as being of statistical significance.

## Discussion

This study did not find any evidence that the use of CRP POCT in RTI (lower RTI, bronchitis, chest/respiratory infection and cough) leads to a statistically significant decrease in the total antibiotic prescribing rate in adults older than 18 and younger than 65 years in practices with high antibiotic prescribing rates. However, it did find evidence of a clinically important reduction in total antibiotic prescriptions administered during the trial in several intervention practices; in consultations where the diagnoses mentioned cough, intervention practices had an estimated 21% reduction in the odds of prescribing, and this was increased to 31% in the three high-fidelity practices.

The study found that even in these high-antibiotic-prescribing practices, there were only a small number of consultations and patients who had acute cough as their main symptom and therefore benefitted from CRP POCT. Our data indicate that in these high-prescribing practices, CRP POCTs were not used in line with NICE guidance as about half of the eligible patients received immediate antibiotics rather than delayed antibiotic prescriptions and may have been using the tests outside the recommended indications. Diagnostic scores are useful tools as a higher diagnostic score was associated with fewer patients with a CRP reading of < 20 mg/L.

Our study confirmed that patients were generally happy about CRP POCT, reporting that the tests can give clinical staff a better basis for treatment decisions, and that the finger prick should be of little concern.

A main strength of our study is that the practices involved were non-research practices in the usual NHS non-trial setting. This means that the patient views were of routine general practice, providing a true representation of the current pressures CCG and the NHS face today. 

While only one CCG with an ethnically diverse patient population was included in the study, which may compromise the representativeness for the whole UK, we took every effort that a range of practices, patients and general practice staff were included in the study. This study’s sample reflects an example of England NHS, with varying acceptance and use of diagnostic tools. Practices varied in size and methods of implementation. Main users of the machine included GPs, prescribing pharmacists and practice nurses, reflecting the real environment of POCT in routine general practice and the variety of staff involved. More patients were involved in this present study than in the other research practice-based studies [17], reflecting the true behaviour in a busy service with high prescribing.

Given the considerable variation in prescribing between practices, the study sample size would need to be about four times larger to provide sufficient statistical power to detect a relative reduction in the odds of dispensing of 0.88, which equates to an absolute 5% reduction for the observed levels of dispensing. It should be noted that as high-prescribing practices were included in the study, they would have reduced prescribing because of the regression to the mean; however this has been considered by including data from intervention and control practices both before and after the trial.

A further limitation is that the EMIS data are only as reliable as the data that are inputted by clinicians.

It should be considered that it was impossible to blind practices to the intervention to use CRP POCT, they knew that their antibiotic use was routinely monitored, would continue to be monitored, and that this was an evaluation to determine if CRP POCT could help reduce antibiotic use in acute cough as part of a national antimicrobial stewardship programme.

An RCT in the Netherlands with 40 GPs from 20 general practices reported that GPs in the CRP test group prescribed significantly fewer antibiotics than in the control group (31% vs 53%; p = 0.02) [15]; our study did not see this significant reduction, using non-research practices and routine general practice. Cals et al. also found that family physicians trained in enhanced communication skills prescribed significantly fewer antibiotics during episodes of RTI in the 3.5 years following the Dutch trial [24], something which our study did not focus on specifically. A communication-based CRP POCT intervention may be better placed in England to attempt to educate patients and increase awareness around antibiotics. Also a recent systematic review including 15 studies across the world, including Denmark, Germany, the Netherlands and Norway, reported that the use of CRP-driven antibiotic therapy was associated with a decreased duration of antibiotic use in neonatal and adult patients [14].

Qualitative interviews and focus groups with the general practice staff involved in the present study was conducted in 2017 and 2018 [18] and support our understanding of existing barriers and facilitators to successful implementation of CRP POCT in routine primary care. 

Our study reports that only 22% of patients with a CRP result between 20 and 100 mg/L were managed in line with NICE guidance to consider a delayed antibiotic prescription. However, no known qualitative or quantitative studies on the diagnosis management of patients with CRP reading of 20–100 mg/L have been published to understand why treatment is not managed in line with NICE guidance. Previous research reported that general practice staff are familiar with CRP POCT NICE guidance but some would prefer to use clinical judgement and be safe and prescribe [18].

A multi-country study in research practices across Europe found that almost all patients would be happy to be managed with the addition of a POCT for lower RTI and patients with experience of POCT accepted it as part of routine care [25]. Our study adds patient views that CRP POCT aid clinical diagnosis, provided quick results and reduced unnecessary antibiotic use. Another European study reported that most patients who received a CRP POCT were satisfied with their consultation although many did not receive an antibiotic [26]. Patient feedback was also positive in a small study in Wales which supports patients’ views in our study that CRP POCT was useful, convenient and comfortable [17].

In the Nordic countries and Switzerland, trained staff undertake diagnostic tests in the GP offices and there is no extra work or cost for GPs when requesting a CRP POC test [27,28]. Under such conditions, implementing CRP POCT is no problem. However, CRP testing is more difficult when the clinician or other practice staff have to undertake the POCT themselves. 

Primary care commissioners are those who work for the CCG to directly commission primary medical services and performance manage practices, in the UK. The variability in use of CRP testing in line with NICE guidance indicates that national and local guidance, and training on the use and interpretation of CRP POCT, needs to be clear and readily available for general practice staff in CCG considering using the test. As there were limited opportunities to use CRP POCT across practices, the machines will be most beneficial in larger GP practices with more patients. More work is needed in the group of patients with intermediate CRP results of 20–100 mg/L to establish how management of these patients in line with NICE guidance could be attained; learning from other European studies would be helpful.

Adopting CRP POCT into routine care in the UK needs a clear CCG and practice action plan, guidance, training and an individual who sees most patients eligible for a CRP POCT.

Practice managers, general practice staff and commissioners are all influenced by the cost of diagnostic tools. Economic evaluations show cost-effectiveness of CRP POCT over existing management of RTI in primary care [28]. However, the upfront costs to general practices still needs to be established. It would be useful to evaluate CRP POCTs in larger practices (> 20,000 patients) for feasibility, efficiency and cost-effectiveness. 

Our study identified examples showing that is it feasible for practices to adopt CRP POCT into routine general practice in line with O’Neill’s suggestion that a test should be mandatory before an antibiotic is prescribed [7], and their success should be shared with other CCG.

## Supplementary Data

147000 Supplementary Material

## References

[1] Public Health England (PHE). English surveillance programme for antimicrobial utilisation and resistance (ESPAUR). Report 2018. London: PHE; 2018. Available from: https://allcatsrgrey.org.uk/wp/download/public_health/ESPAUR_2018_report.pdf

[2] National Institute for Health and Care Excellence (NICE). Respiratory tract infections (self-limiting): prescribing antibiotics. Clinical guideline [CG69]. London: NICE; 23 Jul 2008. Available from: https://www.nice.org.uk/guidance/cg69 31815394

[3] Smieszek T Pouwels KB Dolk FCK Smith DRM Hopkins S Sharland M Potential for reducing inappropriate antibiotic prescribing in English primary care. J Antimicrob Chemother. 2018;73(suppl_2):ii36-43. 10.1093/jac/dkx500 29490058 PMC5890667

[4] Little P Stuart B Moore M Coenen S Butler CC Godycki-Cwirko M Amoxicillin for acute lower-respiratory-tract infection in primary care when pneumonia is not suspected: a 12-country, randomised, placebo-controlled trial. Lancet Infect Dis. 2013;13(2):123-9. 10.1016/S1473-3099(12)70300-6 23265995

[5] Smith SM Fahey T Smucny J Becker LA Antibiotics for acute bronchitis. Cochrane Database Syst Rev. 2014;3(3):CD000245. 10.1002/14651858.CD000245.pub3 24585130

[6] Department of Health. UK five-year antimicrobial resistance strategy 2013 to 2018. London: Department of Health; 2013. Available from: https://www. gov.uk/ government/ uploads/system/ uploads/ attachment_ data/ file/ 244058/ 20130902_ UK_ 5_ year_AMR_ strategy.pdf

[7] O’Niell J. 2016. Review on antimicrobial resistance. London: UK Department of Health; May 2016. Available from: https://amr-review.org/sites/default/files/160518_Final%20paper_with%20cover.pdf

[8] National Institute for Health and Care Excellence (NICE). Pneumonia in adults: diagnosis and management. Clinical guideline [CG191]. London: NICE; 2014.Available from: https://www.nice.org.uk/guidance/cg191/resources/pneumonia-in-adults-diagnosis-and-management-35109868127173 31841289

[9] National Institute for Health and Care Excellence (NICE). Cough (acute): antimicrobial prescribing. London: NICE; Feb 2019. Available from: https://www.nice.org.uk/guidance/ng120/resources/visual-summary-pdf-6664861405 31769946

[10] Oppong R Coast J Hood K Nuttall J Smith RD Butler CC Resource use and costs of treating acute cough/lower respiratory tract infections in 13 European countries: results and challenges. Eur J Health Econ. 2011;12(4):319-29. 10.1007/s10198-010-0239-1 20364288

[11] Huang Y Chen R Wu T Wei X Guo A Association between point-of-care CRP testing and antibiotic prescribing in respiratory tract infections: a systematic review and meta-analysis of primary care studies. Br J Gen Pract. 2013;63(616):e787-94. 10.3399/bjgp13X674477 24267862 PMC3809432

[12] Joshi A Perin DP Gehle A Nsiah-Kumi PA Feasibility of using C-reactive protein for point-of-care testing. Technol Health Care. 2013;21(3):233-40. 10.3233/THC-130720 23792796

[13] Engel MF Paling FP Hoepelman AI van der Meer V Oosterheert JJ Evaluating the evidence for the implementation of C-reactive protein measurement in adult patients with suspected lower respiratory tract infection in primary care: a systematic review. Fam Pract. 2012;29(4):383-93. 10.1093/fampra/cmr119 22159030

[14] Petel D Winters N Gore GC Papenburg J Beltempo M Lacroix J Use of C-reactive protein to tailor antibiotic use: a systematic review and meta-analysis. BMJ Open. 2018;8(12):e022133. 10.1136/bmjopen-2018-022133 30580258 PMC6318522

[15] Cals JW Butler CC Hopstaken RM Hood K Dinant GJ Effect of point of care testing for C reactive protein and training in communication skills on antibiotic use in lower respiratory tract infections: cluster randomised trial. BMJ. 2009;338(may05 1):b1374. 10.1136/bmj.b1374 19416992 PMC2677640

[16] Cals JW Chappin FH Hopstaken RM van Leeuwen ME Hood K Butler CC C-reactive protein point-of-care testing for lower respiratory tract infections: a qualitative evaluation of experiences by GPs. Fam Pract. 2010;27(2):212-8. 10.1093/fampra/cmp088 20022909

[17] Hughes A Gwyn L Harris S Clarke C Evaluating a point-of-care C reactive protein test to support antibiotic prescribing decisions in a general practice. Clin Pharm. 2016;8(10):309-8.

[18] Eley CV Sharma A Lecky DM Lee H McNulty CAM Qualitative study to explore the views of general practice staff on the use of point-of-care C reactive protein testing for the management of lower respiratory tract infections in routine general practice in England. BMJ Open. 2018;8(10):e023925. 10.1136/bmjopen-2018-023925 30361406 PMC6224729

[19] McNulty C Ricketts EJ Rugman C Hogan A Charlett A Campbell R A qualitative study exploring the acceptability of the McNulty-Zelen design for randomised controlled trials evaluating educational interventions. BMC Fam Pract. 2015;16(69):169. 10.1186/s12875-015-0356-0 26577832 PMC4647292

[20] National Institute for Health and Care Excellence (NICE). Alere Afinion CRP for C-reactive protein testing in primary care. London: NICE; 14 Sep 2016. Available from: https://www.nice.org.uk/advice/mib81/resources/alere-afinion-crp-for-creactive-protein-testing-in-primary-care-pdf-63499402887109

[21] Ward C Point-of-care C-reactive protein testing to optimise antibiotic use in a primary care urgent care centre setting. BMJ Open Qual. 2018;7(4):e000391. 10.1136/bmjoq-2018-000391 30397661 PMC6203029

[22] Abbott. Alere Afinion CRP. Warrington: Abbott. [Accessed: Sep 2018]. Available from: https://www.alere.com/en/home/product-details/afinion-crp.html

[23] NHS Health Research Authority. Is my study research?. London: NHS Health Research Authority. [Accessed: 28 Apr 2017]. Available from: http://www.hra.nhs.uk/documents/2016/06/defining-research.pdf

[24] Cals JWL de Bock L Beckers PJHW Francis NA Hopstaken RM Hood K Enhanced communication skills and C-reactive protein point-of-care testing for respiratory tract infection: 3.5-year follow-up of a cluster randomized trial. Ann Fam Med. 2013;11(2):157-64. 10.1370/afm.1477 23508603 PMC3601394

[25] Wood F Brookes-Howell L Hood K Cooper L Verheij T Goossens H A multi-country qualitative study of clinicians’ and patients’ views on point of care tests for lower respiratory tract infection. Fam Pract. 2011;28(6):661-9. 10.1093/fampra/cmr031 21653924

[26] Tonkin-Crine S Anthierens S Francis NA Brugman C Fernandez-Vandellos P Krawczyk J Exploring patients’ views of primary care consultations with contrasting interventions for acute cough: a six-country European qualitative study. NPJ Prim Care Respir Med. 2014;24(1):14026. 10.1038/npjpcrm.2014.26 25030621 PMC4373386

[27] Rebnord IK Hunskaar S Gjesdal S Hetlevik Ø Point-of-care testing with CRP in primary care: a registry-based observational study from Norway. BMC Fam Pract. 2015;16(170):170. 10.1186/s12875-015-0385-8 26585447 PMC4653870

[28] Cooke J Butler C Hopstaken R Dryden MS McNulty C Hurding S Narrative review of primary care point-of-care testing (POCT) and antibacterial use in respiratory tract infection (RTI). BMJ Open Respir Res. 2015;2(1):e000086. 10.1136/bmjresp-2015-000086 25973210 PMC4426285

