# Supplementary material for: Effects of primary care C-reactive protein point-of-care testing on antibiotic prescribing by general practice staff: pragmatic randomised controlled trial, England, 2016 and 2017
Source: Euro Surveill. 2020 Nov 5;25(44):1900408. doi: 10.2807/1560-7917.ES.2020.25.44.1900408 (PMC7645970; doi:10.2807/1560-7917.ES.2020.25.44.1900408)
Supplement: Supplementary Material [file 1900408_ELEY_Supplement.pdf]

This supplementary material is hosted by Eurosurveillance as supporting information alongside the article *'Effects of primary care C-reactive protein point-of-care testing on antibiotic prescribing by general practice staff: pragmatic randomised controlled trial, England, 2016 and 2017'*, on behalf of the authors, who remain responsible for the accuracy and appropriateness of the content. The same standards for ethics, copyright, attributions and permissions as for the article apply. Supplements are not edited by Eurosurveillance and the journal is not responsible for the maintenance of any links or email addresses provided therein.

Supplement S1
CRP Testing - Patient Questionnaire

EMIS Number: xxxxxxx

You went to your doctors on the *date* about a cough and may have had a finger prick blood test.

1. **Did you have a finger prick blood test when you went to your doctors for a cough?**

   YES / NO (please circle)

If you answered YES to the question above please answer these questions too by circling your answer:

2. **Did you feel the explanation of the purpose of the test was?**

   Very good, satisfactory, poor, no explanation was given

3. **Please tell us how comfortable you found the test?**

   Very uncomfortable Comfortable Very comfortable

4. **Please tell us how convenient you found the test?**

   Very inconvenient Convenient Very convenient

5. **Please tell us how useful you think the test was?**

   Very useful Useful Not sure Not at all

6. **Would you recommend that others with cough should have this test?**

   Yes most definitely Maybe Not sure Never

7. **Would you expect a test next time you have a cough?**

   Definitely yes depends on my symptoms, No

8. **Would you accept the test next time you saw your GP with a cough if it was offered to you?**

   Yes No Not sure

9. Who performed the test?

10. How long did you feel the test prolonged your practice visit?

5, 10, 15, 20, 30, 45, 60 minutes.

11. Would you be happy for this test to be done at a pharmacy local to you?

Yes No Not sure

12. Please tell us what was good about having the test done?

13. Please tell us what you did not like about having the test done?

14. Please tell us about any other comments you may have about your consultation or the test in this box

15. Please can you indicate your gender?

Male Female

16. Please can you tell us your age?

17. Please can you tell us the name of your general practice?

Thank you for answering this questionnaire.
Please return your completed questionnaire to us in the enclosed envelope.
